# Novel clinical and genetic insight into *CXorf56*-associated intellectual disability

**DOI:** 10.1038/s41431-019-0558-3

**Published:** 2019-12-10

**Authors:** Maria Eugenia Rocha, Tainá Regina Damaceno Silveira, Erina Sasaki, Daíse Moreno Sás, Charles Marques Lourenço, Krishna K. Kandaswamy, Christian Beetz, Arndt Rolfs, Peter Bauer, Willie Reardon, Aida M. Bertoli-Avella

**Affiliations:** 1 Centogene AG, Rostock, Germany; 2 Clinical Genetics, Children’s Health Ireland (CHI), Crumlin, Ireland; 3 Genotyping, Diagnóstico Genéticos, Botucatu, São Paulo Brazil; 4 Faculdade de Medicina, Centro Universitario Estácio de Ribeirão Preto, Ribeirão Preto, São Paulo Brazil

**Keywords:** Genetics research, Neurodevelopmental disorders, Genetics of the nervous system

## Abstract

Intellectual disability (ID) is one of most frequent reasons for genetic consultation. The complex molecular anatomy of ID ranges from complete chromosomal imbalances to single nucleotide variant changes occurring de novo, with thousands of genes identified. This extreme genetic heterogeneity challenges the molecular diagnosis, which mostly requires a genomic approach. *CXorf56* is largely uncharacterized and was recently proposed as a candidate ID gene based on findings in a single Dutch family. Here, we describe nine cases (six males and three females) from three unrelated families. Exome sequencing and combined database analyses, identified family-specific *CXorf56* variants (NM_022101.3:c.498_503del, p.(Glu167_Glu168del) and c.303_304delCTinsACCC, p.(Phe101Leufs*20)) that segregated with the ID phenotype. These variants are presumably leading to loss-of-function, which is the proposed disease mechanism. Clinically, *CXorf56-*related disease is a slowly progressive neurological disorder. The phenotype is more severe in hemizygote males, but might also manifests in heterozygote females, which showed skewed X-inactivation patterns in blood. Male patients might present previously unreported neurological features such as epilepsy, abnormal gait, tremor, and clonus, which extends the clinical spectrum of the disorder. In conclusion, we confirm the causative role of variants in *CXorf56* for an X-linked form of intellectual disability with additional neurological features. The gene should be considered for molecular diagnostics of patients with ID, specifically when family history is suggestive of X-linked inheritance. Further work is needed to understand the role of this gene in neurodevelopment and intellectual disability.

## Introduction

Intellectual disability (ID) is a neurodevelopmental disorder affecting 1% of the population worldwide. It is characterized by significant limitations in intellectual functioning (reasoning, learning, problem solving) and adaptive behavior (conceptual, social, and practical skills), which manifest before the age of 18 years [1]. A large proportion of IDs have known genetic etiology [2–4], which suggests that an ID phenotype can emerge from many different pathomechanisms.

Recently, Verkerk et al. [5], described *CXorf56* as a new candidate gene for ID in a large family with mild X-linked ID (four males and one female). These five patients presented with nonsyndromic ID and behavioral issues. Males relatives were notably affected, while a female carrier presented a milder phenotype, consistent with a common presentation of X-linked disorders. Via linkage analysis followed by whole genome sequencing, the authors uncovered a frameshift variant in exon 2 of *CXorf56* (NM_022101.3c.159_160insTA) that leads to nonsense-mediated decay (NMD) with reduced mRNA expression in the affected individuals. No other patients with *CXorf56* causative variants were identified among 413 unrelated individuals with ID without specific diagnosis [5].

Establishing the molecular diagnosis for a patient with ID is relevant for clinical management, genetic counseling and determining recurrence risks in the family. Currently, first-tier diagnostic tests in patients with ID are exome and genome sequencing (WES/WGS) and chromosomal microarray analysis (CMA) [6]. By WES, in a diagnostic setting, we identified two additional disease-causing variants in *CXorf56*. This article describes the clinical and genetic features of three unrelated families, expanding the related phenotype and confirming the causative role of *CXorf56* variants in a nonsyndromic form of ID.

## Materials and methods

### Patients

All genetic analyses were performed in concordance to the provisions of the German Gene Diagnostic Act (Gendiagnostikgesetz), and informed written consent was obtained from patients’ parents and referring clinicians, including consent for publication of patients’ images (two families).

### Whole exome sequencing (WES)

Patient blood samples were referred for routine genetic diagnostic workup by experienced clinical geneticists. DNA was extracted by standard methods. WES was performed as described previously [7]. In short, the Nextera Rapid Capture Exome Kit (Illumina, San Diego, CA) or the SureSelect Human All Exon kit (Agilent, Santa Clara, CA) were used for enrichment, and a HiSeq4000 (Illumina) instrument for the actual sequencing with the average coverage targeted to 100×. Variants calling, annotation and prioritization was based on a set of publicly available and in house developed tools. WES was performed in index, parents and affected nephew from family 1 (I-1, I-2, II-8, III-1), index case from family 2 (II-2) and index case and parents from family 3.

The variant-containing exons of *CXorf56* (NM_022101.3) were amplified (primers available upon request) and Sanger-sequenced from both sides on a 3730xl sequencer (Thermo Fisher Scientific, Waltham, MA) in all available family members for co-segregation analysis. Exon numbering was used according to reference sequence NM_022101.3 (exons 1–7).

Detected *CXorf56* variants have been submitted to the Leiden Open Variation database, http://www.lovd.nl/CXorf56 (Individual IDs: 00266162 and 00266164).

### X-inactivation studies

**Fig. 1.**
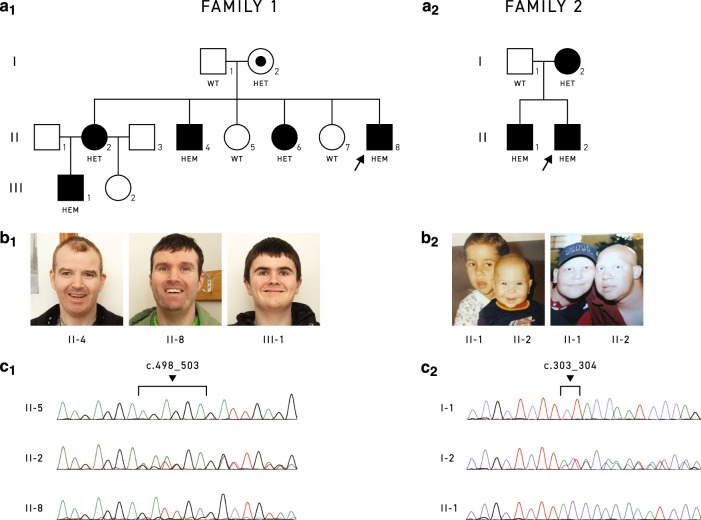
Family trees, facial photographs and *CXorf56* detected variants in Family 1 and 2. **a** Genealogical trees of family 1 and 2 suggesting an X-linked mode of inheritance. Genotype of the individuals related to *CXorf56* variant is shown below each symbol. **b** Photographs of patients from (b_**1**_) family 1, II-4, II-8, III-1 (from left to right, ages 39, 31, and 16 years old). No dysmorphic features were observed. (b_**2**_) Family 2, male patients at different ages. Left panel II-1 (6 years old) and II-2 (1 year old). Right panel: II-1 (22 years old) and II-2 (16 years old). Mild dysmorphic features are noted. Absence of scalp hair and eyebrows can be noted in right image. **c** Sanger traces showing the corresponding CXorf56 region. (**c**_1_) Family 1, exon 5 NM_022101.3:c.498_503del, p.(Glu167_Glu168del) and (**c**_2_) Family 2, exon 4, (NM_022101.3:c.303_304delCTinsACCC, p.(Phe101Leufs*20). From top to bottom: WT wild type, HET heterozygote, HEM hemizygote).

X-inactivation status was investigated by the ‘Rare & Inherited disease genomic laboratory’ in London, UK, using blood samples from three female individuals from family 1 (I-2, II-2, II-6, Fig. 1a_1_). For the study, polymorphic markers, which are differently methylated on the inactive and active X-chromosome, were used. The androgen receptor (AR) was studied by fluorescent PCR in the presence or absence of the methylation sensitive enzyme *Hpa*II as previously described [8].

## Results

### Clinical findings

In family 1 from Ireland, there are three males and two females affected with mild to severe ID, consistent with an X-linked inheritance pattern (Fig. 1a_1_). The mother (I-2) of the patients is a 68 years old female, her cognitive status is known as normal, she is a retired nurse.

II-2 is a 41 years old female with mild ID, occipital frontal circumference (OFC) in the 90–97th percentile. She is not dysmorphic, and does not present tremor or epilepsy. Despite attending a special needs school, she did not complete her education. She lives independently and takes care of her two children. II-4 (Fig. 1b_1_) is a 39 years old male with severe ID, neurodevelopmental developmental delay (NDD), infancy onset epilepsy, and behavioral issues. His OFC is in the 25–50th percentile. Neurological examination revealed fine tremor in hands and lower limbs, past pointing, clonus, hyperreflexia and abnormal gait. He is not capable of performing self-care activities, and lives in a residential center. II-6 is a 32 years old female with mild learning difficulties. She is not dysmorphic, and has mild fine tremor and brisk reflexes. She attends a day care center and has some independence. II-8 (Fig. 1b_1_) is a 31 years old male with severe ID and epilepsy since infancy, his OFC is in the 90th percentile. In neurological examination, he has brisk deep tendon reflexes and sustained clonus, but no tremor. He attended a special needs school, and is currently overseen at a day care center. He is able to self-care. III-1 (Fig. 1b_1_) is a 16 years old boy with moderate ID, NDD, moderate hearing loss, no tremor and an OFC in the 98th percentile. He has a normal brain MRI. He attends a special needs school.

Family 2 (Fig. 1a_2_) originates from Brazil. The mother (I-2) is 51 years old and has alopecia areata that developed at age 20. She finished regular education but with noted learning difficulties; for instance, she was unable to perform simple math operations. She had no seizures. She had never had an employment, and suffers from depression. Her OFC is in the 50th percentile. Her neurological examination was unremarkable; no dysmorphic features were observed. She had normal brain MRI.

The index II-2 (Fig. 1b_2_) is a 22-year-old male, who is more severely affected than his older brother. There were no relevant obstetric or perinatal findings. He presented with NDD and later neurodevelopmental regression after epileptic crises (focal seizures) onset at the age of 12 years. Seizures are partially responsive to pharmacological treatment (using three anticonvulsants: topiramate, clobazam, and valproic acid). Since childhood, he has demonstrated aggressive behavior with agitation, been diagnosed with attention deficit hyperactivity disorder (ADHD). He was able to attend a regular school with additional support but performed poorly, he could not finish elementary studies. As comorbidities, patient has persistent increased levels of prolactin, gastroesophageal reflux disease with hiatal hernia and alopecia areata. His first brain MRI (with 12 years of age) was normal. His second brain MRI, at age 22, only showed a slight brain atrophy; hearing studies were normal. Currently, he has moderate ID associated with progressive gait difficulties. His OFC is in the 90th percentile. Clinical examination revealed mild dysmorphic features (elongated face, long philtrum, prominent ears, bulbous nose, hypoplastic columella), fine tremor in hands and lower limbs, hyperreflexia and a wide-based gait.

His older brother II-1 has milder ID, with no seizures and alopecia. He had bilateral hydronephrosis due to ureterocele and was born with bilateral cryptorchidism and a left inguinal hernia (all surgically corrected). He attended a regular school with very poor performance. The patient was diagnosed with ADHD since childhood. He has a normal brain MRI and normal hearing studies. Clinical examination revealed OFC in the 90th percentile, similar dysmorphic features to his brother, fine tremor in hands, and hyperreflexia. He is able to perform an unskilled job. Both brothers started to lose hair from age of 10–12 years and presented alopecia from 16 to 18 years (Fig. 1b_2_).

### Family 3

The clinical phenotype of the index case is highly similar to family 1 and 2, with ID as main feature. Unfortunately, no additional clinical details can be provided.

### Genetic findings

Given the positive family history, a genetic cause of ID was considered likely in both families. Genetic workup in a routine diagnostic setting was therefore requested.

In family 1, X-linked inheritance was suspected. For II-8 and III-1, CMA, and *FMR1* tests were normal. For III-1, additional karyotyping results were unremarkable. WES was performed in an extended trio setting (index, both parents, affected nephew) on I-1, I-2, II-8, and III-1. The only plausible candidate shared by the patients was a variant in exon 5 of *CXorf56* (NM_022101.3:c.498_503del, p.(Glu167_Glu168del)). This is an in-frame deletion of 6 bps, which causes the loss of two amino acid residues. This protein region is fully conserved across species (Supplementary figure). Sanger sequencing as performed in nine available family members revealed presence of the variant in all affected individuals as well as in female I-2. Unaffected female siblings did not carry the variant (II-5, II-7, Fig. 1a_1_, c_1_).

We then searched our in house database CentoMD^®^ [9] for rare variants in *CXorf56*. We identified a patient (family 2, II-2) with a hemizygous *CXorf56* insertion-deletion variant in exon 4 leading to a frameshift (NM_022101.3:c.303_304delCTinsACCC, p.(Phe101Leufs*20)). In this patient, an X-linked form of ID was suspected given two affected males including the index (Fig. 1a_2_), however, *CXorf56* was not known as an ID candidate gene, at the time of the initial WES analysis. Sanger sequencing of all available family members confirmed co-segregation of the variant (Fig. 1a_2_, c_2_). No relevant variants were identified in the WES data of II-2 that could explain the alopecia.

A third family was identified in our database with the same *CXorf56* variant detected in family 1. The index is hemizygote for the NM_022101.3:c.498_503del variant, which occurred de novo (WES data).

Both variants were absent from public databases, and detected for the first time in the CentoMD^®^ database. According to ACMG guidelines, the variant NM_022101.3:c.498_503del, p.(Glu167_Glu168del) (family 1 and 3) was classified as likely pathogenic, given the following criteria: absence from control databases, protein length change as a result of in-frame deletion and co-segregation with the disease in several family members [10]. The variant identified in family 2 (NM_022101.3:c.303_304delCTinsACCC, p.(Phe101Leufs*20)) was similarly classified as likely pathogenic given the criteria: absence from control databases and LoF type variant (frameshift) in a gene where LoF is a known mechanism of disease [10].

### X-inactivation analysis in family 1

Skewing of X-inactivation in lymphocytes was shown for two female cases from family 1: 97% of the putative carrier chromosomes were inactivated for II-2, and 83% for II-6. In addition, for the asymptomatic female carrier I-2, a nonrandom X-inactivation pattern was detected, there was inactivation of all putative carrier X-chromosomes analyzed.

## Discussion

By WES and combined database analyses, we identified two novel disease-causing variants in the *CXorf56* gene in three unrelated families with nine cases of X-linked, nonsyndromic ID.

All male patients presented with a slowly progressive neurodevelopmental disorder characterized by an initial NDD evolving to mild or severe ID and selected additional features. These included: abnormal reflexes (four cases), fine tremor (four cases), seizures (three cases), clonus (two cases), and abnormal gait (two cases), extending the phenotypic spectrum of the disorder. This indicates that *CXorf56-*related disease extends beyond ID to a more complex neurological disorder. Follow-up of several patients from childhood to adulthood indicates this is a slowly progressive neurodevelopmental disorder.

As alopecia was present only in patients from family 2, it is currently not clear whether this is part of the same neurological phenotype. The combination of ID and alopecia has been reported before [11], but inherited as an autosomal recessive disorder [12]. Both Reza Sailani et al. [13] and Besnard et al. [14] have recently reported a rare neuroectodermal syndrome with total or partial absence of hair and variable ID. Most of the patients in those studies presented with congenital alopecia, in contrast, individuals from family 2 of our study developed alopecia later in their teens. No relevant variants that could explain alopecia were detected in WES data from individual II-2 (family 2).

The three female cases reported here had mild intellectual and learning disabilities without major additional features. The previously reported female by Verkerk et al. presented mainly with moderate ID, behavioral issues, and mild dysmorphic features. X-chromosome inactivation analysis did not show a skewed methylation pattern (54%) in the affected female, while six unaffected female carriers showed a 76–93% inactivation of the maternally inherited allele [5]. X-methylation analyses in three females from family 1 (two mildly affected and one unaffected) confirmed skewed X-inactivation in blood cells from mildly symptomatic females (97 and 83%, II-2 and II-6), while 100% skewed X-inactivation pattern of the putative carrier chromosome was observed in the asymptomatic female (I-2). Assuming that the observed skewed X-inactivation pattern at the *AR* is in linkage with *CXorf56* in the family, our data would argue that even a minor reduction in *CXorf56* could be detrimental for normal neurological functioning. Several limitations of the method need to be considered, namely, inactivation status at the *CXorf56* locus might be different from that observed at the *AR* locus, and skewing might vary among different tissues [15]. Therefore, further functional data are needed to confirm our observation.

In our patients, an in-frame deletion of two residues and an insertion–deletion leading to a frameshift and premature stop were identified. Both variants are located more C-terminal (exons 4 and 5) to the previously reported variant by Verkerk et al. (exon 2) [5], which was shown to be subjected to NMD, with loss-of-function as the likely disease mechanism. The newly described variants are inferred to lead to LoF. The gene is defined as highly LoF intolerant in gnomAD, given the high pLI score (0.95, https://gnomad.broadinstitute.org/gene/ENSG00000018610) and the complete absence of individuals with LoF variants from databases (gnomAD, ExAC, Exome variant server, accession date 14 October 2019). Unfortunately, no material was available to perform expression assays for patients in our study.

*CXorf56* is located on chromosome Xq24, in a region where genomic deletions have been reported in patients with syndromic ID. At least eight patients have been published with large Xq24 deletions including *CXorf56* and the neighboring *UBE2A* gene [16–19]. Single nucleotide variants, as well as large deletions within *UBE2A* are known to cause syndromic ID (Nascimento type [20], OMIM 300860). Interestingly, ‘patient 8′ described by Czeschik et al. and patient 371424 (Decipher [21]) have smaller deletions on Xq24 spanning uniquely *CXorf56* and *UBE2A* (X:118679518-118717453 and X:118510482-118725778). Both cases present with syndromic ID including congenital heart defects, short stature, hypertelorism, and other dysmorphic features [16, 21]. Phenotypically, these patients seem similar to patients with larger deletions or with smaller *UBE2A-* restricted variants. In-depth genotype–phenotype correlation analysis is warranted in larger series of patients. One additional case is reported in Decipher with ID and a small deletion including only *CXorf56*, suggesting that in line with our findings, *CXorf56* gene deletions are leading to ID.

Current data indicates that patients with *UBE2A*-related ID are clinically different from cases with variants affecting only *CXorf56*, with the former having a syndromic type of ID with extra-neurological features and recognizable dysmorphism.

Little is known about the function of the gene/protein. CXorf56 protein is expressed in the nucleus and cell soma of most neurons throughout the brain cortex and cerebellum in 8-week-old wild-type mice [5] and it has been shown to have a relevant role during brain development in mice [22]. Additional information on gene expression is shown in several other tissues according to several databases (https://genevisible.com/tissues/HS/UniProt/Q9H5V9, https://bgee.org/?page=gene&gene_id=ENSG00000018610 and https://gtexportal.org/home/gene/CXORF56#geneExpression). This might suggest a role of the gene beyond the nervous system.

Although descriptions of additional patients are necessary for further delineation of the *CXorf56*-related disease, the above identified patients show that this is a slowly progressive neurodevelopmental disorder with ID and additional neurological features manifesting mainly in males. More importantly, our data confirm the causative role of *CXorf56*. The gene should be considered for molecular diagnostics of patients with ID, specifically in those whose family history is suggestive of X-linked ID. Further work is needed to understand the role of this gene in neurodevelopment and ID.

## Supplementary information

Supplementary Figure. CXorf56 protein conservation in the area affected by variant NM_022101.3:c.498_503del, p.(Glu167_Glu168del)

Supplementary Figure Legend
