# Supplementary material for: Novel clinical and genetic insight into CXorf56-associated intellectual disability
Source: Eur J Hum Genet. 2019 Dec 10;28(3):367–72. doi: 10.1038/s41431-019-0558-3 (PMC7028711; doi:10.1038/s41431-019-0558-3)
Supplement: Supplementary file 2 — Supplementary Figure Legend [file 41431_2019_558_MOESM2_ESM.docx]

Supplementary Figure. CXorf56 protein conservation in the area affected by variant NM_022101.3:c.498_503del, p.(Glu167_Glu168del).
